# Virtual resident showcase: leveraging an institutional repository during COVID-19 social distancing

**DOI:** 10.5195/jmla.2020.1052

**Published:** 2020-10-01

**Authors:** Heather J. Martin, Amanda Schwartz

**Affiliations:** 1 Heather.Martin@Providence.org, Director, System Library Services, Providence St. Joseph Health, Portland, OR; 2 amanda.schwartz@providence.org, Digital Asset Librarian, System Library Services, Providence St. Joseph Health, Missoula, MT

## Abstract

While institutional repositories are common in medical schools and academic health centers, they have been used by only a small number of health systems to track and promote their research and scholarly activity. This article describes how Providence System Library Services leveraged their existing institutional repository platform to substitute a virtual showcase for an annual in-person event.

While institutional repositories (IRs) are common in medical schools and academic health centers [1], they have been used by only a small number of health systems [2] to track and promote their research and scholarly activity. This article describes how Providence System Library Services leveraged their existing IR platform to substitute a virtual showcase for an annual in-person event.

The Providence Digital Commons was launched in July of 2018. The repository uses BePress Digital Commons, a hosted software product that allows customizable metadata, repository architecture, and collection of institutional materials into specific research topic groups. The Providence Digital Commons has been able to collect and organize institutional scholarly works into multiple collections, giving visibility to the unique facets of research and clinical excellence in a large health care organization [3].

Looking to engage the Providence Oregon Graduate Medical Education (GME) department with the IR and highlight resident scholarly activity, library leadership offered to host an online space in tandem with the Providence Oregon GME Academic Achievement Day, an annual in-person poster conference. The virtual showcase would be able to preserve posters for viewing after the event, with the added benefit of citation tracking and statistics for residents to view the impact of their research. This feature, which uses PlumX altmetrics, is included as part of the BePress platform with no additional fee or set-up. A stable permanent uniform resource locator (URL) for individual posters could be used by residents during their job hunts, and program leadership could use the collection for recruitment and Accreditation Council for Graduate Medical Education (ACGME) reporting.

Before library staff secured a consensus to move forward on the collaboration, early 2020 brought the COVID-19 global pandemic, and all in-person events were indefinitely postponed. This delay enabled the library to finally gain unanimous buy-in from the GME department, nimbly design a metadata architecture, and organize the submission of posters from eight different programs. Resident research and quality improvement projects could now be shared more widely than ever before.

The Providence Oregon Academic Achievement Day collection was launched on April 29, 2020. In the first month, the collection has seen 620 downloads and 969 metadata hits by users from 47 countries. Initial reactions from the GME program leadership have been positive, and plans are already in the works to add a virtual component to next year's in-person event. In the first month, individual residency programs handled all promotion of the collection. While user engagement has been strong, future campaigns will benefit from a coordinated marketing campaign.

BePress's flexible platform allowed last minute changes and late submissions. Architecture was redesigned to include an additional fellowship program, and the layout changed to show graphic thumbnails rather than text-heavy metadata. The structure is designed to easily accommodate additional GME programs across the system in the coming years. Future plans include integrating a video or audio component, as BePress allows both hosted and embedded multimedia streaming. Library staff are exploring technology solutions with the vendor as well as with the internal information technology (IT) department to allow some posters to be hosted behind a firewall to protect potentially identifiable case information.

This pilot of a virtual event and collection—sparked by library innovation during a pandemic—exemplifies the successful melding of poster sessions and IR collections into a virtual and widely accessible showcase. This model could be adopted by both health care systems and academic libraries that are looking to build an online component into a traditional conference model.
